# The Importance of Social Support, Positive Identity, and Resilience in the Successful Aging of Older Sexual Minority Men

**DOI:** 10.3390/geriatrics6040098

**Published:** 2021-10-10

**Authors:** Henrique Pereira, Patrícia Silva

**Affiliations:** 1 Department of Psychology and Education, Faculty of Social and Human Sciences, University of Beira Interior, Pólo IV, 6200-209 Covilhã, Portugal; pg.silva@ubi.pt; 2 Research Centre in Sports Sciences, Health Sciences and Human Development (CIDESD), 5001-801 Vila Real, Portugal

**Keywords:** aging, older gay men, older bisexual men, social support, resilience, positive identity, successful aging, healthy aging, Portugal

## Abstract

The aim of this study was to assess the relationship between social support, positive identity, and resilience and the successful aging of older sexual minority men. The study involved having 210 self-identified gay and bisexual men aged between 50 and 80 years complete a cross-sectional online survey comprised of sociodemographic information; the Portuguese version of the Multidimensional Scale of Perceived Social Support; the Lesbian, Gay, and multifactor Bisexual Positive Identity Measure; the Connor–Davidson Resilience Scale 10; and the Successful Aging Perceptions Scale. The results showed that self-identified gay participants showed higher levels of positive identity, while bisexual participants scored higher for resilience, mental health, and successful aging. Higher levels of social support, resilience, and positive identity were significant predictors of mental health (28%), physical health (18%), and successful aging (10%) in our sample. These results offer similarities with the growing body of literature on the positive factors of successful aging in the gay and bisexual men communities, which is an important step in the development of aging and health preventive initiatives among this population.

## 1. Introduction

Gay and bisexual men often experience extensive psychosocial vulnerabilities, mainly due to exposure to social stigma [1,2], and thus become more likely to develop physical and mental health problems and greater psychological distress and loneliness, in addition to financial, social, and professional vulnerabilities [3,4,5]. According to the minority stress theory [6,7], these experiences are directly related to long-term discrimination and stigmatization that are present in an oppressive environment, characterized by heteronormativity.

For older sexual minority men (OSMM), in addition to stigma associated with their sexual orientation status, they also face stigma associated with aging related to changes in physical functioning and social roles, since older individuals are seen as no longer contributing to the development of society; this creates increased feelings of loneliness, helplessness, devaluation, and low self-esteem [8,9,10]. Ultimately, this double stigma will impair their successful aging [11,12].

It is also important to recall that OSMM grew up at a time when homosexuality and bisexuality were severely criminalized and considered mental health disorders [13,14]. Before 1973, the American Psychiatric Association classified homosexuality as a sociopathic personality disorder [15,16]. As a direct effect of this oppressive environment, many OSMM had to hide their sexual identity or lead a concealed life, living in constant fear of rejection or persecution [17,18], which is associated with worse health outcomes and unsuccessful aging [14,15].

Although there is no consensus on the definition of successful aging among sexual minorities, this process seems to encompass a subjective and dynamic system of acceptance of their positive gay/bisexual identity, with positive integration of mental, physical, and emotional health specificities. These factors would then be shaped by internal components, such as personality, coping skills, optimism, and resilience competence, as developed during the adversities experienced as a sexual minority [19]; and external components, such as social support networks, involvement in social activities, and specific policies aimed at protecting and reducing discrimination of sexual minorities [20,21,22].

In this sense, although problems experienced by individuals who are part of a sexual minority can cause extensive negative impacts, they can also contribute to the development of resilience, strengths, and skills that assist in positive coping during the aging process [22,23]. Older sexual minority individuals seem to be more resilient, more courageous, and exhibit a positive sense of identity and an optimistic attitude on life [19,24,25].

Although resilience is not easy to define, and is addressed from different perspectives in the literature, such as a personality trait or the influence of personality traits and coping experiences [26], it is robust in protecting mental health, and is directly related to the multifaceted process of accepting a positive identity as a sexual minority. A study by Bruce et al. [27] with sexual minority youth found that the stigma and stress of concealing their sexual identity were related to the later development of a positive identity, suggesting that these adversities may have served as positive resources for this developmental task. Positive identity has also been associated with better physical and mental health outcomes [6,7,28,29]; greater social contacts with family, friends, intimate partners, and the LGBT community; activism for social justice; self-awareness; authenticity; and self-intimacy [30,31,32]. In Portugal, OSMM who had a more positive gay/bisexual identity and stronger characteristics common to aging were the ones who felt most comfortable with themselves [33].

Maintaining a secure social support network is especially important in the process of accepting a positive identity and successful aging among OSMM. Maintaining significant relationships, a network of friends, and family support help with stress management [9,34] and are associated with good physical and mental health results, as well as overall satisfaction with life [15,24,35]. Hence, sexual identity acceptance enables a more positive aging process [36]. On the other hand, lower levels of social relationships were predictors of greater vulnerability among Portuguese OSMM; namely, depressive symptoms, loneliness, and poor quality of life [2,12,37,38].

Contact with the LGBT community and benefiting from social policies are also related to increased resilience, positive acceptance, sense of belonging, social protection, and support among sexual minorities [35,39,40]. Being involved in community matters is associated with higher levels of general wellbeing [28,41], in addition to reduced impacts from loneliness and psychosocial problems [38,40]. This is especially important among OSMM, as they are more likely to be single and have a smaller support network than the rest of the LGBT population [42,43,44].

In Portugal, the current political achievements of support and inclusion collaborate so that the country is one of seven European countries with the greatest respect for equality for LGBT people [45]. Measures such as the legalization of same-sex marriage and the adoption of children by same-sex couples, in addition to specific legislation to combat discrimination against people on the basis of sexual orientation, aim to accelerate the inclusion of these people into the society they live in [37,46].

Notwithstanding these achievements, social attitudes in Portugal are still characterized by sexual stigma, sexual prejudice, and sexual discrimination, mainly due to conservative attitudes shaped by religious and heterosexist norms that affect quality of life for sexual minority individuals [33,47]. In addition, despite the efforts of Portuguese researchers, there are still few studies on OSMM in that country. Therefore, the objective of this study was to assess the levels of social support, positive identity, and resilience, and their relationship with successful aging among OSMM. This information is fundamental for the construction of effective measures to support positive aging in this population in Portugal.

## 2. Materials and Methods

### 2.1. Participants

A group of 210 men between 50 and 80 years of age participated in this study (mean age = 60.03; SD = 6.33). Of the participants, 85.3% identified as gay and 14.7% as bisexual. The majority claimed to be employed (68.7%), possess a university education (68.6%), and be of middle socioeconomic status (68.7%). Regarding family status, 51.7% said they were single, 51.2% lived alone, and 68.7% had no children. Table 1 presents the sample’s sociodemographic characteristics in further detail.

### 2.2. Measurement Instruments

The study collected sociodemographic information using a questionnaire that asked participants about their age, sexual orientation, profession, socioeconomic status, educational level, marital status, living situation, and if they had children.

The study also utilized the Portuguese version of the Multidimensional Scale of Perceived Social Support, or MSPSS [48], to measure participants’ social support, which was originally developed by Zimet et al. [49] and is one of the most extensively used instruments to assess social support. It assesses subjective perceptions of social support by collecting perceived adequacy of social support from three sources: family (e.g., “I can talk about my problems with my family”), friends (e.g., “I have friends with whom I can share my joys and sorrows”), and significant others (e.g., “There is a special person in my life who cares about my feelings”). It is a Likert-type self-response scale, with 12 items and 7 response categories with a response scale between 1 (completely disagree) and 7 (completely agree). In the current sample, Cronbach’s α for the scale was 0.95, demonstrating excellent internal reliability.

The multifactor Lesbian, Gay, and Bisexual Positive Identity Measure (LGB-PIM) [31], adapted to Portuguese, was used to measure positive identity, which consists of 25 items and is composed of five dimensions: self-awareness (e.g., “My LGBT identity motivates me to be more self-aware”), authenticity (e.g., “I embrace my LGBT identity”), community (e.g., “I feel included in the LGBT community”), intimacy (e.g., “My LGBT identity allows me to understand my sexual partner better”), and social justice (e.g., “I am more sensitive to prejudice and discrimination against others because of my LGBT identity”). Respondents rated each item on a seven-point scale ranging from 1 (strongly disagree) to 7 (strongly agree). An average score was calculated for all items, where a higher score indicated greater positive identity. In the current sample, Cronbach’s α for the LGB-PIM was 0.92, demonstrating excellent internal reliability.

This study measured participants’ resilience using the Connor–Davidson Resilience Scale 10 (CDRISC-10) [50], which consists of 10 items assessed using a Likert scale ranging from 1 to 5 points. The CDRISC-10 asks subjects to evaluate statements and the extent to which they applied to the subjects’ lives over the past month (e.g., “Under pressure I stay focused” or “I think of myself as a strong person when facing challenges”). Resilience levels were evaluated based on the total sum of all items of this self-response scale, with higher scores reflecting higher levels of resilience. As in the Portuguese validation study [51], in the current sample, the Cronbach’s α for the CDRISC-10 was 0.93, demonstrating excellent internal reliability.

Finally, this study measured participants’ successful aging perceptions using the Portuguese version of the Successful Aging Perceptions Scale (SAPS) [52]. Respondents were asked to rate how important (ranging from 1 = not at all important to 5 = very important) they thought of 12 successful elements of aging (being independent, having friends, living with children or other adult family members, financial security, having physical mobility, being free of chronic diseases, being able to work, being cared for by children or other family members, having an active social life, living with a partner, being able to take care of family members, and being happy). In the current sample, Cronbach’s α for the SAPS was 0.86, demonstrating very good internal reliability. In addition to responding to the SAPS, participants were asked to respond to questions concerning the self-assessment of their physical and mental health, using a Likert-type response scale ranging from 1 (very poor) to 5 (excellent). Participants responded to the following questions: “In general, would you say your physical health is…” and “In general, would you say your mental health is…”.

### 2.3. Procedures

This study created a website that included the measurement instruments, which was later disseminated through the Internet. The researchers specifically created this website for the purposes of this study, and participants were invited to voluntarily partake in the study through a message containing a link to the website. Sample recruitment was conducted among gay and bisexual men over 50 years of age, using social networks, LGBT organizations, mailing lists, and electronic notifications. Although most developed world countries characterize old age starting at 60 years and above, we included middle-aged men (>50) in the sample because this stage of life is distinctive in both the quantity of stressors experienced and their nature, and we wanted to capture men’s ageing complexities in relation to midlife as the aging cycle begins at this phase. The study recruitment message directed participants to the website, which explained the study objectives, the anonymous and confidential nature of its personal data collection, informed consent, and a guarantee that the data collected would be used solely for statistical purposes, in accordance with the Declaration of Helsinki concerning the ethical principles for research involving human subjects. Ethical approval was granted by the Ethics Committee of the University of Beira Interior (code number CEUBI-Pj-2020-088).

## 3. Results

Table 2 shows the results for participants’ levels of social support, positive identity, resilience, physical and mental health, and successful aging by sexual orientation. Participants’ general mental health perceptions were also relatively high, as shown by an average score of 4.18 (SD = 0.93) on the general mental functioning self-assessment. The sample demonstrated moderate scores for all variables, but when analyzing differences by sexual orientation (gay and bisexual), the following results showed statistically significant differences (*p* < 0.05): positive identity (t_(208)_ = 3.104; *p* = 0.002), indicating that gay participants scored higher; resilience (t_(207)_ = −2.515; *p* = 0.013), indicating that bisexual participants scored higher; mental health self-assessment (t_(207)_ = −2.250; *p* = 0.029), indicating that bisexual participants scored higher; and successful aging (t_(208)_ = −1.948; *p* = 0.048), indicating that bisexual participants scored higher (see Table 2). Sample size discrepancy (gay participants = 179 vs. bisexual participants = 31) may be attributable to the fact that bisexual older men are far less likely than older gay men to be “out”, and, therefore, are more invisible and harder to reach.

A correlation matrix was created using all variables to assess the levels of association among social support, positive identity, resilience, mental and physical health, and successful aging perception variables. As displayed in Table 3, significant correlations were found between mental health self-assessment and social support (*r* = 0.391; *p* < 0.001), positive identity (*r* = 0.286; *p* < 0.05), and resilience (*r* = 0.475; *p* < 0.001); physical health self-assessment and social support (*r* = 0.330; *p* < 0.001), resilience (*r* = 0.347; *p* < 0.001), and mental health (*r* = 0.393; *p* < 0.001); and successful aging and social support (*r* = 0.308; *p* < 0.001), and physical health self-assessment (*r* = 0.179; *p* < 0.001).

Finally, three multiple linear regressions were performed to determine the predictive effect of social support, positive identity, and resilience on successful aging perceptions, mental health, and physical health. The following assumptions were met: linearity (the relationship between independent variables and the mean of dependent variable was linear), homoscedasticity (the variance of residual was the same for any value of any independent variable), independence (observations were independent of each other), and normality (for any fixed value of any independent variable, the dependent was normally distributed. To measure multicollinearity, we used the variance inflation factor (VIF = 1), which indicated that the variables were not correlated. As shown in Table 4, all models obtained were significant, indicating that social support, positive identity, and resilience explains 10% of successful aging perceptions (social support being the most significant predictor), 28% of mental health (social support and resilience being the most significant predictors), and 18% of physical health (social support, positive identity, and resilience all being significant predictors).

## 4. Discussion

The main purpose of this research was to assess the relationship between social support, positive identity, and resilience and the successful aging of older gay and bisexual men in Portugal. Our results corroborate other research that aims to address the positive aspects of aging among LGBT people [11,15]. In fact, approaches that focus on the risks for the older LGBT population can lead them to be portrayed as pathological victims, while positive qualities developed by this population, such as building resilience and crisis competence, can go unnoticed [53].

Our results may then be associated with the skills and competencies that these men have learned to deal with the challenges and adversities imposed as a gay or a bisexual man throughout their lives in the context of a heteronormative society that classified them as inappropriate and illegal [14,18]. As a result, older gay and bisexual men have developed coping skills that prepared them to also deal with the specificities of aging, contributing to a better experience of this period of their lives [54,55] compared to their heterosexual peers [39,53].

Gay men showed the greatest positive identity scores. These results were expected to the extent that bisexual men tend to experience greater discrimination and biphobia, both by heterosexual and gay individuals who often point out that these people do not come out as gay [11,56,57]. These attitudes influence a higher level of internalized homonegativity, resulting in less acceptance of their bi identity [58]. Higher levels of positive identity in gay people have been previously highlighted in comparison to their bisexual peers [59,60,61,62,63].

On the other hand, higher levels of resilience, mental health, and successful aging were noted in the bisexual men in the sample. These results may be related to greater learning and crisis resolution skills that these men may have developed because they live in scenarios and contexts of adversity throughout their lives regarding their double stigma exposure [55,58,64] and its possibility of multiple relational experience [65,66,67,68]. In addition, higher levels of resilience [19,69] and positive mental health functioning [19,38] can enhance successful aging among older self-identified bisexual Portuguese men.

For social support and physical health, there were no significant differences between sexual orientations, being moderately above average for the entire sample. Although these results are positive, they contradict most studies that suggest bisexual people have a greater social support network [38], since bisexual men are more likely to have had opposite-sex marital relationships and may also have children [68,70]. In contrast, older gay men tend to have higher rates of loneliness and lack of contact with friends and the LGBT community [43,44], which has already been associated with poorer physical and mental health and overall quality of life, thus compromising successful aging [38,71,72,73].

In relation to physical health, we found studies that point to bisexual men as the most engaged and with the best physical health outcomes [15,24]. In these studies, social engagement on the part of bisexual men, and greater isolation on the part of gay men, were determining factors of the results for physical health. In addition, our results may have been influenced by the sample’s sociodemographic characteristics, since the majority had an academic background, were employed, and had a medium to high socioeconomic status, characteristics that have already been associated with more successful aging [21,74].

Several determinants of successful aging among OSMM have been identified, such as maintaining social contacts, having less perceived loneliness, and better quality of life [15,75,76]. More than half of the sample were single, living alone, and had no children, which may have been reflected in the homogeneity of the results on social support throughout the sample.

Finally, our linear regressions demonstrated that all study variables were significant predictors of mental health (28%), physical health (18%), and successful aging (10%), with social support being more representative in all of them, followed by resilience and positive identity. These results were expected, due to the positive correlation that we found among all study variables. The results also confirm our research hypothesis about the importance of maintaining these positive factors for more successful aging, thus further corroborating the findings of other studies [24,25,39] and pointing to the need to address these issues in gay and bisexual men’s aging strategies.

### 4.1. Limitations and Future Directions

However, this study also has limitations that do not allow for generalization of the results. First, this was a highly differentiated convenience sample, since most participants were employed, educated, single, lived alone, and had no children; thus, they were not representative of the broader Portuguese OSMM population. In addition, we did not control those sociodemographic variables in the regression models, and therefore extraneous factors may be interfering statistically with the results. Future studies should be conducted to control their effects on the dependent variables under study. Second, the majority of the sample self-identified as gay (85.3%), indicating a heterogeneity in the study groups. Third, the survey was disseminated over the Internet through social networks, which may have led to selection bias.

Future studies could benefit from larger and more representative samples, in addition to using other forms of dissemination, such as face-to-face interviews, which could reach other types of participants not included in our sample. Studies that evaluate other aspects, such as socioeconomic characteristics and their impacts on successful aging, could yield a current profile of successfully aging OSMM. Finally, longitudinal studies could assess aging over time, since the aging process is subjective and dynamic and can change according to life experiences [20].

### 4.2. Conclusions and Implications

Despite these limitations, this study provides important contributions to the understanding of successful aging mechanisms among OSMM. These results indicate that, despite the diverse adversities that these men face when dealing with agism and homophobia/biphobia, they are also aging well, with a good perception of physical and mental health, resilience, a positive identity, and positive perceived social support.

In this sense, changing the focus to the positive results of the aging of gay and bisexual men can have an important impact on the advancement of theoretical and practical knowledge for actions that strengthen these factors, which is very important to the strengthening of the LGBT community. In addition, clinical implications can also be taken from these results, as interventions for physical and psychological health of sexual minorities are paramount [77,78]. As such, adopting approaches that encourage positive identity formation [79], resilience development [23], and collaborative work with family, friends, and the LGBT community [38,40] would help to promote social inclusion of OSMM. In addition, it is important to make connections both inside and outside the LGBT communities, ensuring the application of public policies, reducing the negative impacts of prejudice, decriminalization, and stigmatization on older gay and bisexual men, and their overall quality of life [80]. Thus, this study makes a fundamental contribution to the promotion the successful aging of OSMM.

## Figures and Tables

**Table 1:** Sociodemographic characteristics (M_age_ = 58.85; SD = 6.33).

		n	%
Sexual Orientation	Gay	179	85.3
	Bisexual	31	14.7
Professional Status	Employed	144	68.7
	Unemployed	20	9.4
	Retired	40	18.9
	Disability recipient	6	3.0
Educational Attainment	Middle school	18	8.5
	High school	48	22.9
	Bachelor’s degree	70	33.3
	Master’s degree	48	22.9
	Doctorate/PhD	26	12.4
Socioeconomic Status	Very low	5	2.5
	Low	20	9.5
	Middle	144	68.7
	High	40	18.9
	Very high	1	0.4
Marital Status	Single	109	51.7
	Dating	22	10.4
	Living with a partner	19	9.0
	De facto same-sex union	14	6.5
	Same-sex marriage	19	9.0
	De facto opposite-sex union	2	1.0
	Opposite-sex marriage	19	9.0
	Divorced/separated	5	2.5
	Widower	2	0.9
Living Situation	Living alone	108	51.2
	Living with a partner	37	17.4
	Living with husband/wife	31	14.9
	Living with children	4	2.0
	Living with parents/father/mother	14	6.5
	Living with friends	9	4.5
	Other	7	3.5
Children	Yes	65	30.8
	No	145	68.7

**Table 2:** Social support, positive identity, resilience, physical and mental health, and successful aging results by sexual orientation.

	Sexual Orientation	M (SD)	t (df)	*p*	Cohen’s d
Social support (1–7)	Gay	4.73 (1.61)	−0.337(204)	0.737	−0.102
	Bisexual	4.83 (1.19)			
Positive identity (1–7)	Gay	4.65 (1.24)	3.104(208)	0.002 *	0.734
	Bisexual	3.91 (1.16)			
Resilience (1–5)	Gay	4.05 (.80)	−2.515(207)	0.013 *	−0.383
	Bisexual	4.43 (.62)			
Physical health self-assessment (1–5)	Gay	3.68 (.78)	−1.742(207)	0.083	−0.260
	Bisexual	3.94 (.72)			
Mental health self-assessment (1–5)	Gay	4.13 (.92)	−2.250(207)	0.029 *	−0.370
	Bisexual	4.50 (.84)			
Successful aging (1–5)	Gay	4.03 (.60)	−1.948(208)	0.048 *	−0.220
	Bisexual	4.24 (.50)			

* *p* < 0.05.

**Table 3:** Correlation values among variables.

	1	2	3	4	5	6
1—Social support	-					
2—Positive identity	0.522 **	-				
3—Resilience	0.421 **	0.286 **	-			
4—Mental health self-assessment	0.391 **	0.160 *	0.475 **	-		
5—Physical health self-assessment	0.330 **	0.063	0.347 **	0.393 **	-	
6—Successful aging	0.308 **	0.095	0.083	0.062	0.179 **	-

* *p* < 0.05; ** *p* < 0.001.

**Table 4:** Social support, positive identity, and resilience linear regression models predicting success aging, mental health, and physical health.

	Successful Aging			Mental Health			Physical Health		
	*B*	*SEB*	*β*	*B*	*SEB*	*β*	*B*	*SEB*	*β*
Social support	0.143	0.031	0.377 **	0.167	0.045	0.279 **	0.155	0.040	0.308 **
Positive identity	−0.039	0.037	−0.083	−0.071	0.052	−0.096	−0.107	0.047	−0.173 *
Resilience	−0.044	0.055	−0.059	0.459	0.079	0.387 **	0.257	0.069	0.263 **
*R* ^2^	0.104			0.277			0.181		
*F for change in R* ^2^	7.782 **			25.641 **			14.788 **		

* *p* < 0.05; ** *p* < 0.001.

## Data Availability

The data presented in this study are available upon request.
